# SCN1A IVS5N+5 G>A Polymorphism and Risk of Febrile Seizure and Epilepsy: A Systematic Review and Meta-Analysis

**DOI:** 10.3389/fneur.2020.581539

**Published:** 2020-12-17

**Authors:** Jindou Hao, Haiying Liu, Jiying Ma, Guosheng Liu, Guoqing Dong, Peihui Liu, Fei Xiao

**Affiliations:** ^1^Department of Paediatrics, The First Affiliated Hospital of Jinan University, Guangzhou, China; ^2^Department of Paediatrics, Affiliated Shenzhen Maternity and Child Healthcare Hospital, Southern Medical University, Shenzhen, China; ^3^Department of Occupational Health Surveillance, Shenzhen Prevention and Treatment Center for Occupational Diseases, Shenzhen, China

**Keywords:** febrile seizure, epilepsy, genetic polymorphisms, risk, meta-analysis

## Abstract

**Background:** Previous studies had investigated the association between polymorphism of IVS5N+5 G>A in *SCN1A* and the risk of febrile seizure and epilepsy. However, the results were inconsistent. We aimed to conduct a systematic review and meta-analysis to evaluate the association between *SCN1A* IVS5N+5 G>A polymorphism and risk of febrile seizures and epilepsy.

**Methods:** We searched Embase, Medline, Scopus, and CNKI for studies on the association between *SCN1A* IVS5N+5 G>A polymorphism and risk of febrile seizures and epilepsy up to 19 February 2020. We pooled odds ratios (ORs) and 95% confidence intervals (CIs) by different genetic models. To explore the source of heterogeneity, we performed the subgroup analysis by ethnicity and source of control.

**Results:** We included a total of 12 studies in the meta-analysis. We found a significant negative association between G allele *SCN1A* IVS5N+5 G>A polymorphism, febrile seizures [G vs. A: OR (95% CI): 0.690 (0.530–0.897); GG vs. AA: 0.503 (0.279–0.908); AG vs. AA: 0.581 (0.460–0.733); GG + AG vs. AA: 0.543 (0.436–0.677); AA + GG vs. AG: 1.309 (1.061–1.615)], and epilepsy [G vs. A: 0.822 (0.750–0.902); GG vs. AA: 0.655 (0.515–0.832); AG vs. AA: 0.780 (0.705–0.862); GG vs. AG + AA: 0.769 (0.625–0.947); GG + AG vs. AA: 0.743 (0.663–0.833); AA + GG vs. AG: 1.093 (1.001–1.193)]. The subgroup analysis shows the association varied by type of disease, ethnicity, and source of control.

**Conclusion:** The present meta-analysis suggests that G allele in *SCN1A* IVS5N+5 G>A polymorphism is a protective factor of febrile seizures and epilepsy. It is possible to determine the vulnerability of each individual to develop febrile seizures or epilepsy genotype by these genetic variants. Future studies with better study designs are needed to confirm the results.

**Study Registration:** This study was registered in the International Prospective register of systematic reviews (PROSPERO, CRD42020163318).

## Introduction

A febrile seizure is a convulsion in a child triggered by fever, usually from a viral infection (1). It often occurs among children aged 0.5 to 5 years (2). There were ~5% of children affected by febrile seizures (3). Its prognosis is generally good, but there is concrete evidence that the affected children have a higher risk for subsequent epilepsy, which is a brain disorder having substantial cognitive, neurobiological, social, and psychological consequences (4). Epilepsy is characterized by an enduring predisposition to generate one or more epileptic seizures (5). It was one of the most commonly seen neurological disorders, affecting about 60 million individuals globally (6). Epilepsy can be fatal if no treatment is given. Patients with epilepsy had higher rates of disability and death (7). Therefore, prevention, early detection, and treatment of febrile seizure and epilepsy are important for child health.

The risk of febrile seizure is closely related to genetics. It was estimated that the heritability was 70% for febrile seizures (8). The role genetics play in the etiology of febrile seizure remains unknown. Recent studies had been conducted in detecting single nucleotide polymorphism (SNP) forms of febrile seizure (9–13). Researchers found that SNPs in *SCN1A*, which is a neuronal voltage-gated sodium channel a1-subunit gene, had a strong association with the risk of febrile seizure and epilepsy (9–13). This sodium channel a1, a type of voltage-gated sodium channels, plays an important role in the central nervous system (14). Dysfunction of this channel will reduce the sodium current in γ-aminobutyric acid and membrane excitability, increasing the risk of febrile seizure and epilepsy (15). Therefore, the SNPs in *SCN1A* appear to be good biomarkers for predicting the risk of febrile seizure and epilepsy among children.

The SNP IVS5N+5 G>A in *SCN1A* can significantly alter the proportions of transcripts of the gene (16). It is located in a donor region of a conserved splice site. The minor allele (G) can influence the 5′ splice donor site of the transcripts of exon 5, which significantly reduces its transcription (17). Evidence indicated the neonatal exon was preferentially expressed in early developmental phases and was upregulated after febrile seizures (18). This SNP has also been found to be associated with an increased risk for epilepsy in children by a similar mechanism, which was influencing the expression level of transcripts of SCN1A and reducing the membrane excitability in the central nervous system (19).

Previous literature had investigated whether the SNP IVS5N+5 G>A in *SCN1A* was associated with the risk of febrile seizure and epilepsy in different studies (9–13). Nevertheless, the results were inconsistent across them. Balan et al. (9) and Schlachter et al. (12) showed an increase in the A-allele and AA-genotype frequencies in patients with febrile seizure or epilepsy compared to the controls, but these current differences did not reach statistical significance in the studies by Le Gal et al. (10), Zhang et al. (13), and Petrovski et al. (11). More recently, some studies also found similar associations for vaccine-proximate febrile seizures and drug-resistant epilepsy (20, 21). Therefore, this study aims to perform a systematic review and meta-analysis on the association between the SNP IVS5N+5 G>A in *SCN1A* and the susceptibility to febrile seizure and epilepsy.

## Methods

### Literature Search

This study was registered in International Prospective register of systematic reviews (PROSPERO, CRD42020163318). This study was conducted according to the MOOSE guideline (22). We conducted electronic searches for all potentially relevant studies that investigate the association for *SCN1A* IVS5N+5 G>A polymorphism and febrile seizure or epilepsy in MEDLINE, EMBASE, SCUPOS, CNKI, and WAN FANG from the databases from the inception till 19 February 2020. Pre-determined search strategies were adopted, including search terms for “*SCN1A*,” “polymorphism,” “febrile,” and “epilepsy.” No restrictions on publication status were imposed. We also searched the reference lists/bibliographies of the included studies manually.

### Inclusion Criteria

To be eligible, studies should include the following features: (i) reporting the relationship between *SCN1A* IVS5N+5 G>A polymorphism and the susceptibility to febrile seizure or epilepsy; (ii) case–control studies as appropriate study design; (iii) focusing on the human subject, and (iv) being published in peer review journals.

### Exclusion Criteria

Studies were excluded for the following reasons: (i) duplicate records; (ii) no reported odds ratio (OR) with the 95% confidence interval; (iii) insufficient data; and (iv) cross-sectional studies, case report or series, reviews, or letters. The reason for excluding cross-sectional studies, case report or series, reviews, or letters was that selected case–control studies performed much better on the casual association between *SCN1A* IVS5N+5 G>A polymorphism and the susceptibility to febrile seizure or epilepsy.

### Literature Selection

We selected eligible studies independently by two reviewers (JH and HL). We screened the title and abstract of the retrieved records from databases and assessed for eligibility. For duplicate or overlapping records, the single most updated and comprehensive version was selected. Disagreements were resolved by discussion, and a consensus was reached between the two reviewers. A third reviewer (GD) was consulted for the unresolved discrepancy.

### Data Extraction

We extracted the following information from each included study with a pre-designed data extraction form: first author, year of publication, country, method, source of control, different subtypes of diseases, the total number of case and control, and the number of cases and controls for AA, AG, and GG. All data were retrieved independently by two reviewers (JH and HL).

### Methodological Quality Assessment

We used the validated Newcastle-Ottawa Scale (NOS) (23) to assess the methodological quality of all included studies with a “star system” to judge three broad perspectives and a total of eight items: (1) adequacy of case definition; (2) representativeness of the cases; (3) selection of controls; (4) definition of controls; (5) comparability cases/controls; (6) ascertainment of the outcome of interest; (7) the same method of ascertainment; (8) non-response rate. A study can be awarded a maximum of nine stars.

### Data Analysis

In this study, we used random-effect model meta-analyses to evaluate the association between *SCN1A* IVS5N+5 G>A polymorphism and the susceptibility to febrile seizure or epilepsy. Pooled ORs with a 95% confidence interval (CI) were calculated for the allelic model (G vs. A), homozygote model (GG vs. AA), heterozygote model (AG vs. AA), recessive (GG vs. AG+AA), dominant model (GG+AG vs. AA), and over-dominant model (AA+GG vs. AG). The reason why the calculation of ORs was based on G allele is that it represented the rare type of allele in comparison to the A allele in most of the studies included (11/12, Table 1). The level of heterogeneity was measured with the *I*^2^ statistic, with *I*^2^ < 25% regarded as low heterogeneity, 25–50% as moderate heterogeneity, and >50% as high heterogeneity. The Hardy–Weinberg equilibrium (HWE) was examined by chi-square or Fisher exact test by using genotype frequencies in the control population (24). All analysis was conducted using Stata 14.0. We conducted a subgroup analysis of patients with epilepsy by stratifying studies based on ethnicity and source of control. *p* < 0.05 was considered as statistically significant. In order to adjust for potential bias generated by multiple comparisons, Bonferroni correction method was used with *p* significant level set at 0.05/20. To examine the potential publication bias caused by the small-study effects, we used funnels plots with Egger's and Begg's tests.

**Table 1:** Characteristics of the included studies.

**Authors**	**Year**	**Country**	**Method**	**SOC**	***P* (HWE)**	**Type**	**Case**				**Control**			
							***n***	**AA**	**AG**	**GG**	***n***	**AA**	**AG**	**GG**
Schlachter	2009	Austria	MAR	PB	0.94	FS only	144	56	72	16	701	187	351	163
						FS + Epi	90	45	35	10	701	187	351	163
						No FS + Epi	486	133	250	103	701	187	351	163
						ALL FS	234	101	107	26	701	187	351	163
						ALL Epi	576	178	285	113	701	187	351	163
Petrovski	2009	Australia	MAR	HB	0.94	FS only	23	10	8	5	701	187	351	163
						FS + Epi	76	27	34	15	701	187	351	163
						No FS + Epi	482	152	218	112	701	187	351	163
						ALL FS	99	37	42	20	701	187	351	163
						ALL Epi	558	179	252	127	701	187	351	163
Zhang	2010	China	MS	PB	0.97	FS + Epi	97	32	53	12	837	279	409	149
						No FS + Epi	625	242	286	97	837	279	409	149
						ALL Epi	722	274	339	109	837	279	409	149
Grover	2010	India	SBE	PB	NA	ALL Epi	362	NA	NA	NA	86	NA	NA	NA
Le Gal	2011	France	PCR	HB	0.04	FS only	102	35	48	19	199	52	113	34
						FS + Epi	62	24	28	10	199	52	113	34
						No FS + Epi	113	36	49	28	199	52	113	34
						ALL FS	164	59	76	29	199	52	113	34
						ALL Epi	175	60	77	38	199	52	113	34
Balan	2012	India	PCR	PB	0.91	FS + Epi	138	55	62	21	282	72	140	70
						No FS + Epi	65	24	35	6	282	72	140	70
						ALL Epi	203	79	97	27	282	72	140	70
Hung	2012	China	RFLP	HB	0.39	ALL Epi	234	90	105	39	189	69	95	25
Kumari	2013	India	PCR	PB	0.86	ALL Epi	485	171	243	71	298	61	146	91
Baum	2014	China	SBE	HB	NA	ALL Epi	819	NA	NA	NA	837	NA	NA	NA
Haerian	2015	China	SBE	HB	0.85	ALL Epi	282	113	130	39	189	167	228	75
		India		HB	0.59	ALL Epi	151	66	66	19	189	87	114	43
		Malaysia		HB	0.55	ALL Epi	243	92	114	37	189	123	169	66
Angelopoulou	2017	Greece	PCR	HB	<0.01	ALL Epi	200	14	153	33	189	10	143	47
Damiano	2019	India	PCR	PB	NA	FS only	144	NA	NA	NA	90	NA	NA	NA

*PCR, polymerase chain reaction; MAR, microarrays; MS, Mass spectrometry; RFLP, polymerase chain reaction-restriction fragment length polymorphism; SBE, single base extension; SOC, source of control; PB, population-based; HB: hospital-based; HWE, Hardy–Weinberg equilibrium; FS, febrile seizure; FS+ Epi, epilepsy with febrile seizure; No FS + Epi, epilepsy without febrile seizure; All FS, all febrile seizure; All Epi, all epilepsy; NA, not aplicable*.

## Results

### Results on Literature Search and Selection

**Figure 1:**
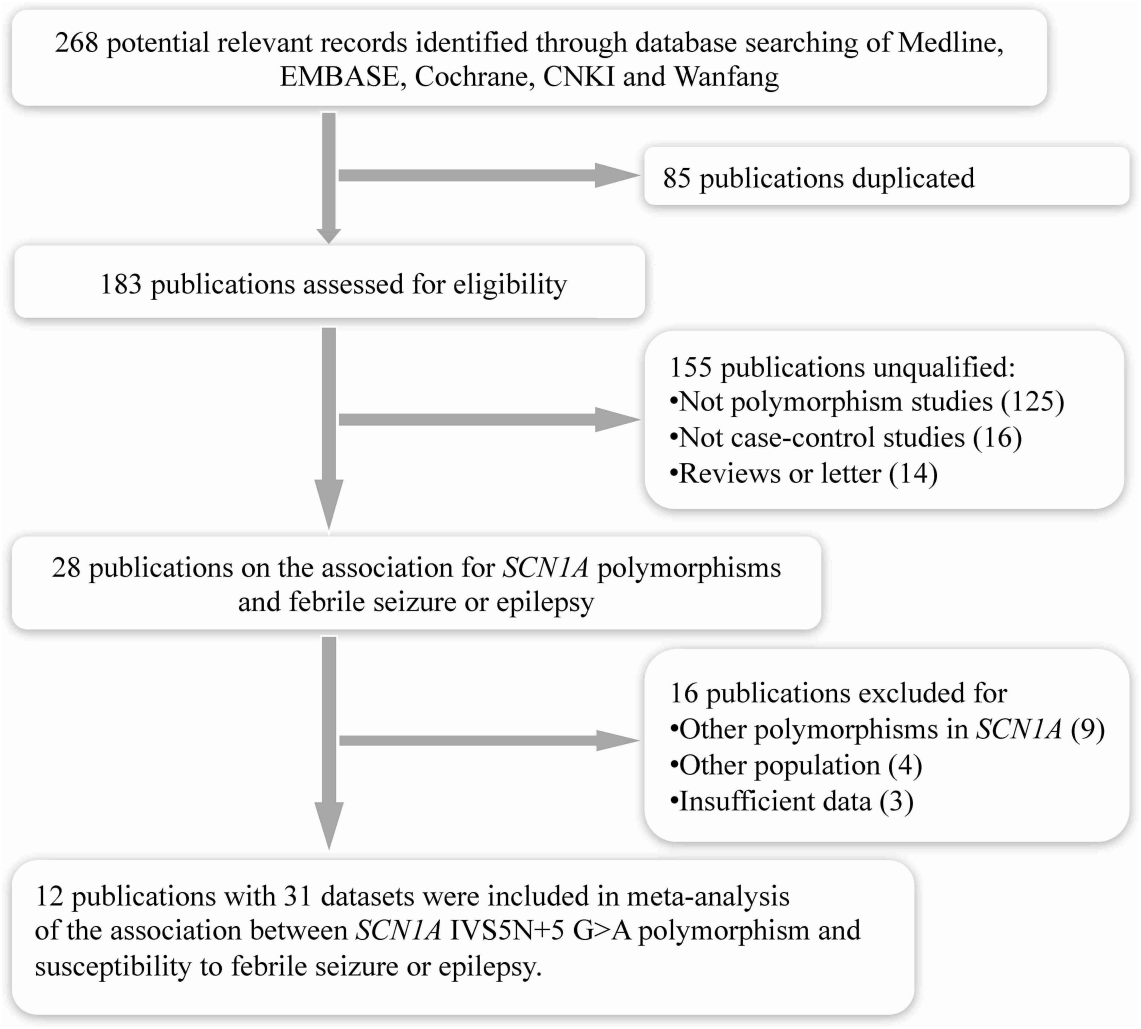
Selection of articles for systematic review.

The search strategy initially generated a total of 268 records from all databases. After removing the duplicate studies (*n* = 85), it included 183 publications assessed for eligibility (Figure 1). After screening titles and abstracts, we excluded 155 publications for they were not polymorphism studies (*n* = 125), not case–control studies (*n* = 16), or reviews and letters (*n* = 14). We retrieved full texts of the remaining 28 for further assessment. Of these, 16 publications were excluded as they were investigating other polymorphisms in SCN1A (*n* = 9), other populations without febrile seizure or epilepsy (*n* = 4), or with insufficient data (*n* = 3). Finally, a total of 12 eligible studies were included in this meta-analysis (9–13, 20, 21, 25–29).

### Study Characteristics

Characteristics of the included studies are summarized in Table 1. The studies were all published in the past 10 years (2009–2019). They were reported from India (5), China (4), Austria (1), Australia (1), France (1), Malaysia (1), and Greece (1). Genotyping was performed using polymerase chain reaction, microarrays, mass spectrometry, polymerase chain reaction-restriction fragment length polymorphism, or single base extension. Controls were from the hospital (*n* = 8) or the general population (*n* = 4). The Hardy–Weinberg equilibrium (HWE) was observed among most of the studies (9/11). The included studies recruited a total of 11,862 participants, with 8,154 cases and 3,708 controls.

### The Methodological Quality of the Included Studies

The overall quality of the 12 studies scored from seven to nine, indicating good quality. All studies had adequacy of case definition, the definition of controls, ascertainment of the outcome of interest, the same method of ascertainment, and low risk in non-response rate. Only one study had an unknown risk of representativeness of the cases. However, half of the studies had an unknown risk of selection of control as they were recruited from the hospital. The comparability cases/controls varied across studies. Details of the methodological quality of the included studies are presented in Table 2.

**Table 2:** Methodological quality of the included studies according to the Newcastle-Ottawa Scale.

**Author**	**Year**	**Adequacy of case definition**	**Representativeness of the Cases**	**Selection of controls**	**Definition of controls**	**Comparability cases/Controls**	**Ascertainment of exposure**	**Same method of ascertainment**	**Non-response rate**
Schlachter	2009	*	*	*	*	**	*	*	*
Petrovski	2009	*	*	NA	*	**	*	*	*
Zhang	2010	*	*	*	*	**	*	*	*
Grover	2010	*	*	*	*	*	*	*	*
Le Gal	2011	*	NA	NA	*	**	*	*	*
Balan	2012	*	*	*	*	**	*	*	*
Hung	2012	*	*	NA	*	*	*	*	*
Kumari	2013	*	*	*	*	*	*	*	*
Baum	2014	*	*	NA	*	**	*	*	*
Haerian	2015	*	*	NA	*	**	*	*	*
Angelopoulou	2017	*	*	NA	*	*	*	*	*
Damiano	2019	*	*	*	*	*	*	*	*

This table identifies “high” quality choices with a “star.” A study can be awarded a maximum of one star for each numbered item within the Selection and Exposure categories. A maximum of two stars can be given for Comparability. *, Yes; NA, not applicable. (http://www.ohri.ca/programs/clinical_epidemiology/oxford.htm).

### Results of Meta-Analysis

**Figure 2:**
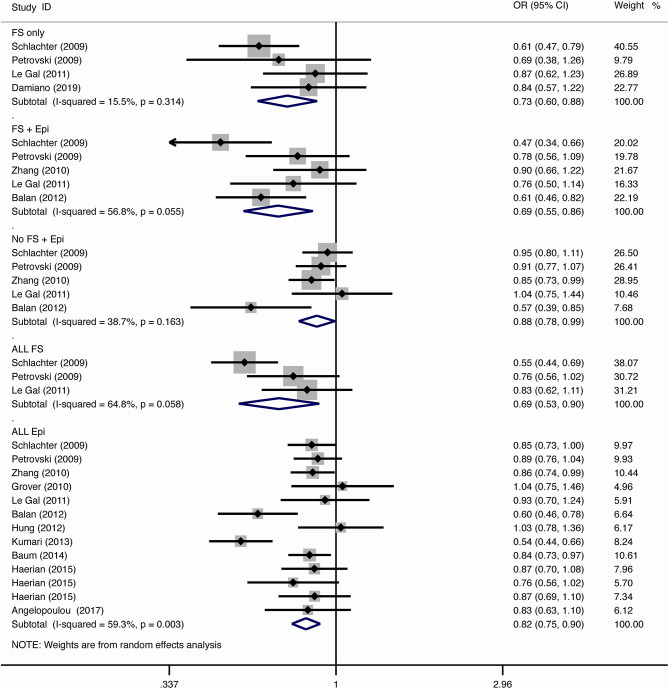
Results of meta-analysis using allelic model (G vs. A).

**Figure 3:**
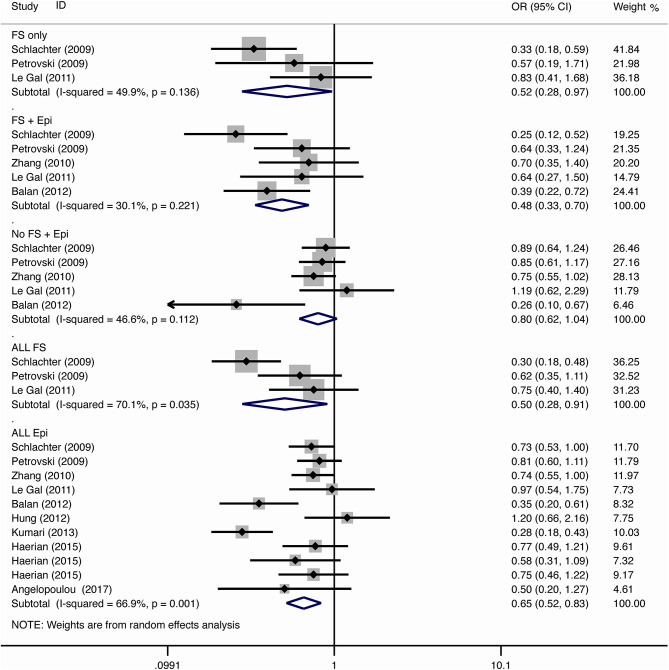
Results of meta-analysis using homozygote model (GG vs. AA).

**Figure 4:**
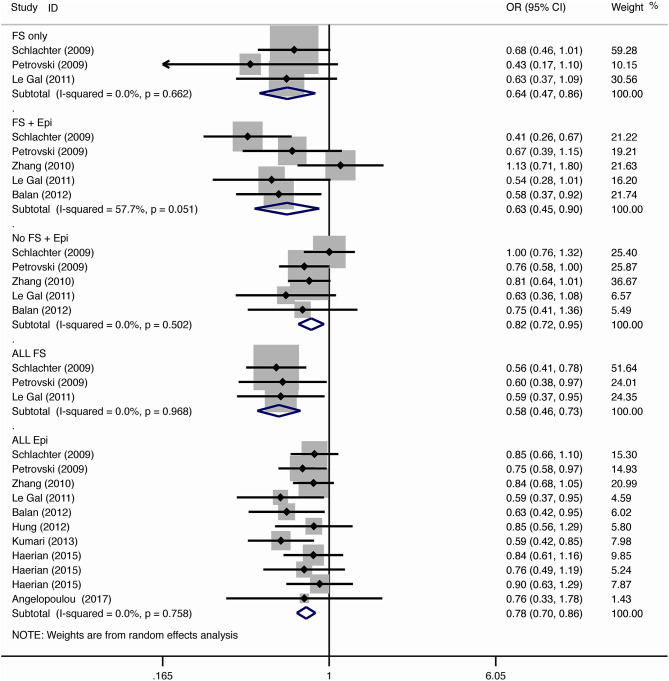
Results of meta-analysis using heterozygote model (AG vs. AA).

**Figure 5:**
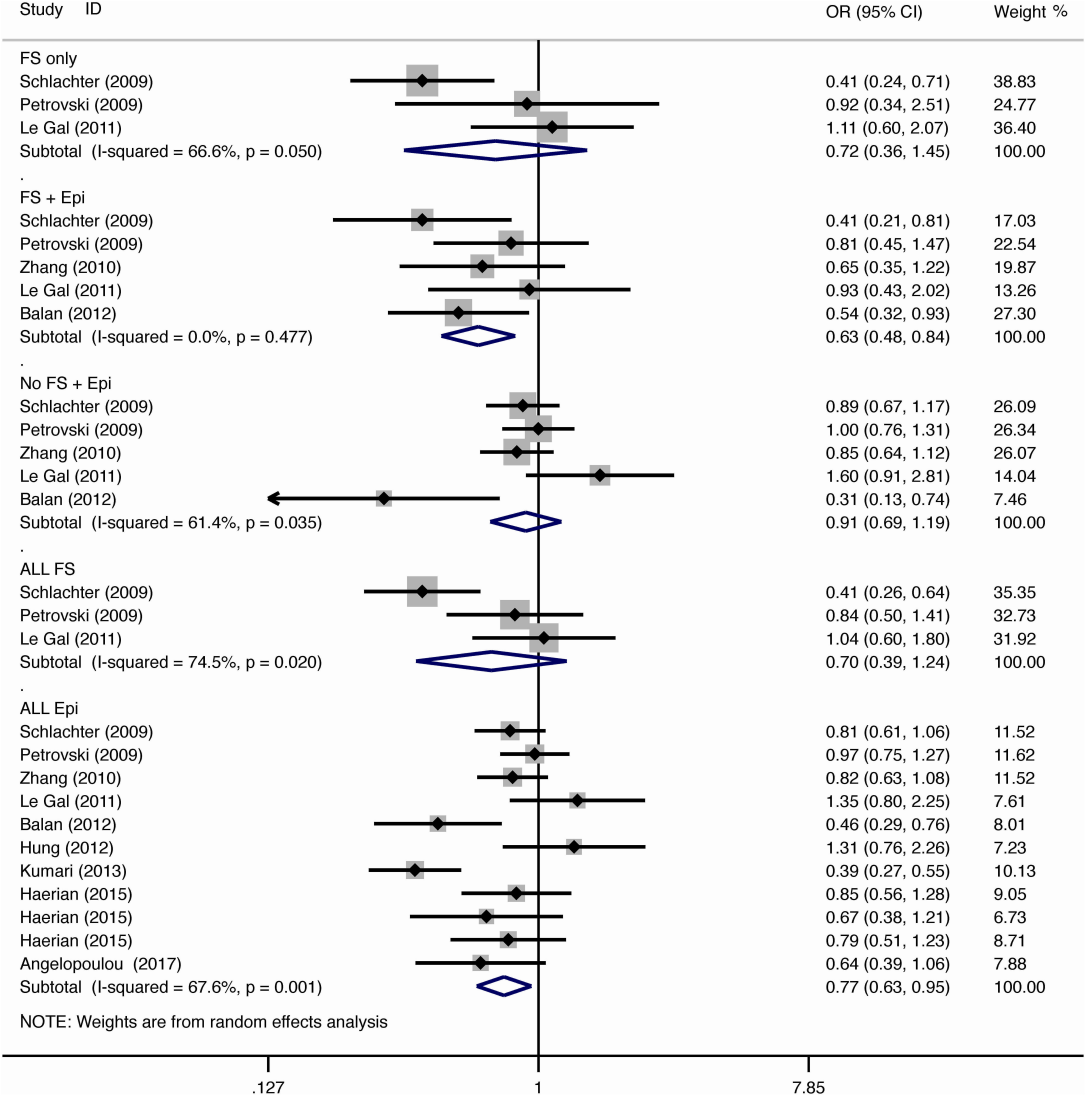
Results of meta-analysis using recessive model (GG vs. AG+AA).

**Figure 6:**
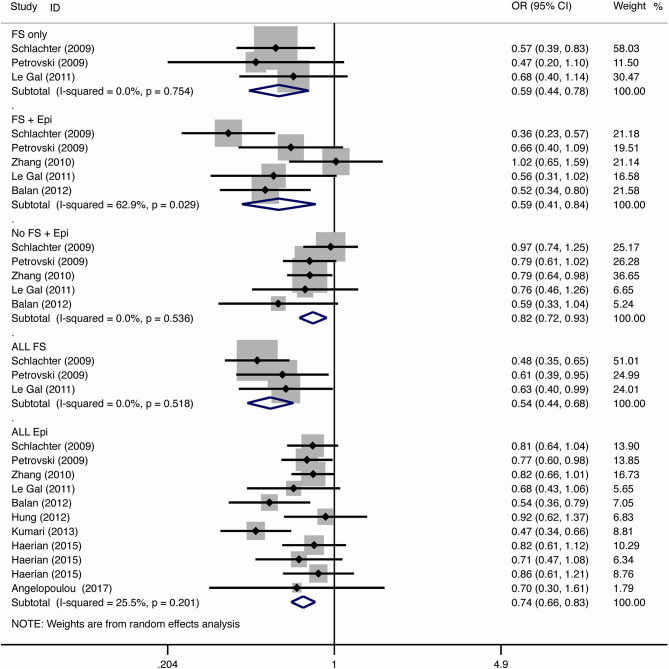
Results of meta-analysis using dominant model (GG+AG vs. AA).

**Figure 7:**
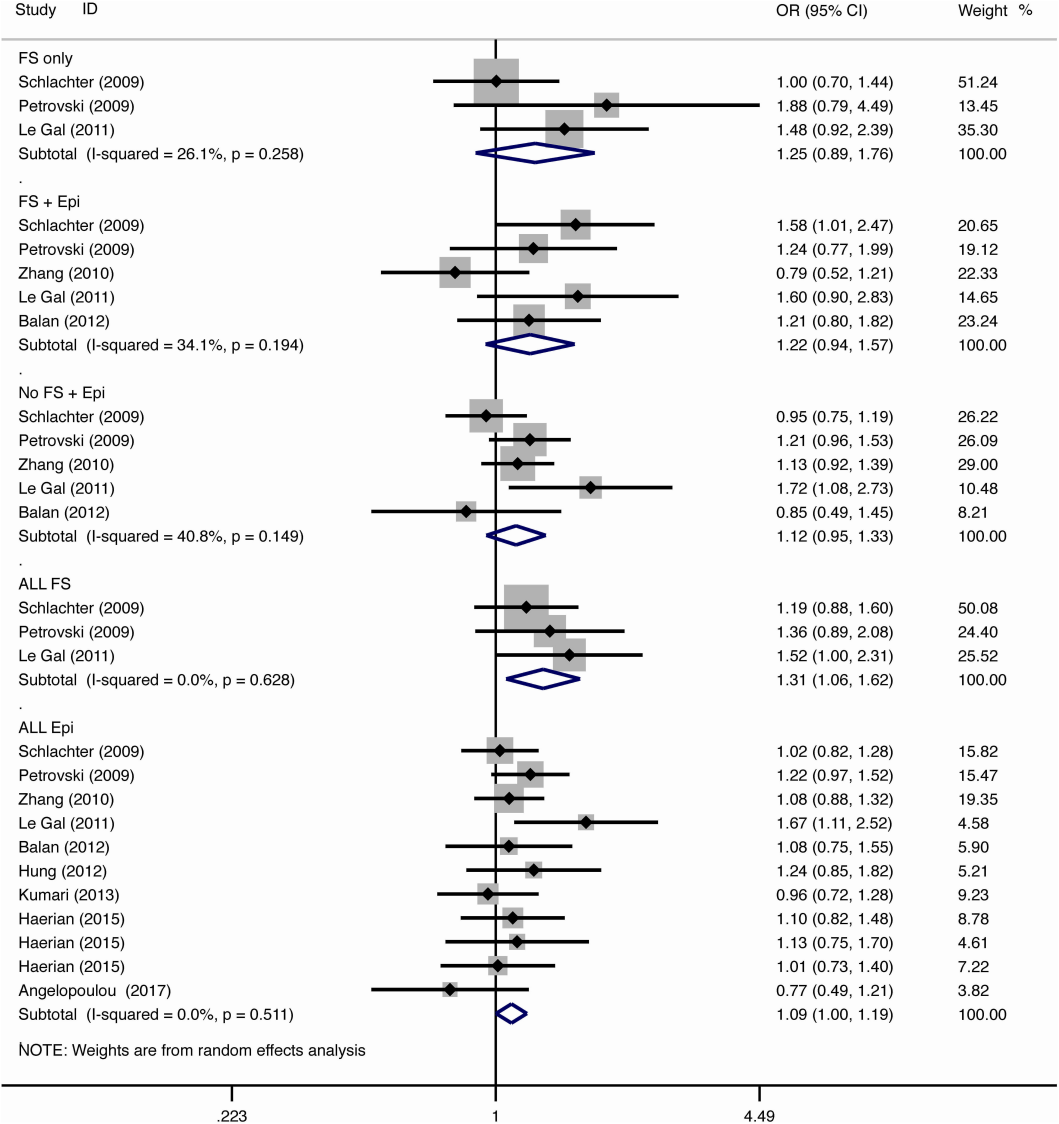
Results of meta-analysis using over-dominant model (AA+GG vs. AG).

The results of meta-analysis using different genetic models were shown in Table 3 and Figures 2–7. We did not identify any publication bias (*p* > 0.05. Supplementary Figures).

**Table 3:** Results of meta-analysis for *SCN1A* IVS5N+5 G>A polymorphism and risk of febrile seizure or epilepsy.

	**Case/Control**	**G vs. A**			**GG vs. AA**			**AG vs. AA**		
		**OR (95% CI)**	***P***	***I*^**2**^ (%)**	**OR (95% CI)**	***P***	***I*^**2**^**	**OR (95% CI)**	***P***	***I*^**2**^**
FS only	413/1,691	0.728 (0.600–0.884)	0.001^**^	15.5	0.519 (0.278–0.969)	0.040^*^	49.8	0.637 (0.471–0.861)	0.003^*^	0
FS + Epi	463/2,720	0.686 (0.547–0.860)	0.001^**^	56.8	0.393 (0.215–0.716)	<0.001^**^	30.1	0.633 (0.446–0.899)	0.011^*^	57.7
No FS + Epi	1,771/2,720	0.882 (0.784–0.993)	0.038^*^	38.7	0.799 (0.615–1.037)	0.091	46.6	0.823 (0.716–0.945)	0.006^*^	0
All FS	497/1,601	0.690 (0.530–0.897)	0.006^*^	64.8	0.503 (0.279–0.908)	0.023^*^	70.1	0.581 (0.460–0.733)	<0.001^**^	0
All Epi	5,010/4,886	0.822 (0.750–0.902)	<0.001^**^	59.3	0.655 (0.515–0.832)	0.001^**^	66.9	0.780 (0.705–0.862)	<0.001^**^	0
Caucasian	1,509/1,790	0.873 (0.792–0.962)	0.006^*^	0	0.775 (0.634–0.949)	0.014^*^	0	0.769 (0.650–0.909)	0.002^**^	0
Chinese	2,057/2,052	0.869 (0.797–0.947)	0.001^**^	0	0.807 (0.640–1.017)	0.069	0.9	0.844 (0.715–0.997)	0.046^*^	0
Indian	1,201/855	0.696 (0.530–0.915)	0.009^*^	75.6	0.366 (0.243–0.552)	<0.001^**^	45.1	0.648 (0.515–0.816)	<0.001^**^	0
PB	2,348/2,204	0.752 (0.607–0.931)	0.009^*^	82	0.494 (0.306–0.800)	0.004^*^	84.4	0.759 (0.638–0.904)	0.002^**^	28.5
HB	2,662/2,682	0.870 (0.807–0.937)	<0.001^**^	0	0.801 (0.665–0.965)	0.020^*^	0	0.786 (0.681–0.907)	0.001^**^	0
	**Case/Control**	**GG vs. AG** **+** **AA**			**GG** **+** **AG vs. AA**			**AA** **+** **GG vs. AG**		
		**OR (95% CI)**	***P***	***I***^**2**^	**OR (95% CI)**	***P***	***I***^**2**^	**OR (95% CI)**	***P***	***I***^**2**^
FS only	413/1,691	0.721 (0.359–1.449)	0.358	66.6	0.589 (0.443–0.783)	<0.001^**^	0	1.252 (0.892–1.756)	0.194	26.1
FS + Epi	463/2,720	0.632 (0.478–0.837)	0.001^**^	0	0.588 (0.414–0.837)	0.003^*^	62.9	1.216 (0.944–1.567)	0.130	34.1
No FS + Epi	1,771/2,720	0.908 (0.693–1.190)	0.486	61.4	0.816 (0.716–0.930)	0.002^**^	0	1.122 (0.948–1.329)	0.181	40.8
All FS	497/1,601	0.699 (0.394–1.241)	0.221	74.5	0.543 (0.436–0.677)	<0.001^**^	0	1.309 (1.061–1.615)	0.012^*^	0
All Epi	5,010/4,886	0.769 (0.625–0.947)	0.013^*^	67.6	0.743 (0.663–0.833)	<0.001^**^	25.5	1.093 (1.001–1.193)	0.046^*^	8
Caucasian	1,509/1,790	0.897 (0.711–1.131)	0.358	40.9	0.773 (0.660–0.905)	0.001^**^	0	1.132 (0.891–1.438)	0.311	60.1
Chinese	2,057/2,052	0.897 (0.708–1.137)	0.370	15.2	0.835 (0.713–0.977)	0.025^*^	0	1.109 (0.953–1.291)	0.180	0
Indian	1,201/855	0.463 (0.345–0.620)	<0.001^**^	19.1	0.553 (0.438–0.699)	<0.001^**^	13.2	1.030 (0.846–1.255)	0.766	0
PB	2,348/2,204	0.604 (0.417–0.876)	0.008^*^	80.2	0.662 (0.508–0.863)	0.002^**^	71.4	1.038 (0.917–1.175)	0.552	0
HB	2,662/2,682	0.911 (0.756–1.097)	0.326	20.9	0.793 (0.693–0.907)	0.001^**^	0	1.148 (0.997–1.322)	0.055	18.4

*FS, febrile seizure; FS+ Epi, epilepsy with febrile seizure; No FS + Epi, epilepsy without febrile seizure; All FS, all febrile seizure; All Epi, all epilepsy; OR, odds ratio; CI, confidence interval; I^2^, 0–25%, means no heterogeneity; 25–50%, means modest heterogeneity; >50%, means high heterogeneity; HB, hospital-based; PB, population-based*;

* *significant at a level of 0.05*;

** *significant after Bonferroni correction (significance was set at 0.05/20)*.

#### Febrile Seizure

For the association between *SCN1A* IVS5N+5 G>A polymorphism and risk of febrile seizure alone, a protective effect was detected in the G allele for allelic model (OR = 0.728, 95%CI: 0.600–0.884, *p* = 0.001, *I*^2^ = 15.5%), homozygote comparison (OR = 0.519, 95%CI: 0.278–0.969, *p* = 0.04, *I*^2^ = 49.8%), heterozygote comparison (OR = 0.637, 95%CI: 0.471–0.861, *p* = 0.003, *I*^2^ = 0%), and dominant model (OR = 0.589, 95%CI: 0.443–0.783, *p* < 0.001, *I*^2^ = 0%), but not in recessive model (OR = 0.721, 95%CI: 0.359–1.449, *p* = 0.358, *I*^2^ = 66.6%) and over-dominant model (OR = 1.252, 95%CI: 0.892–1.756, *p* = 0.194, *I*^2^ = 26.1%). After Bonferroni correction, the associations remained significant for the allelic and dominant models.

For the association between G allele in *SCN1A* IVS5N+5 G>A polymorphism and risk of febrile seizure with epilepsy, a significant negative association was found in the allelic model (OR = 0.686, 95% CI: 0.547–0.860, *p* = 0.001, *I*^2^ = 56.8%), homozygote comparison (OR = 0.393, 95% CI: 0.215–0.716, *p* < 0.001, *I*^2^ = 30.1%), heterozygote comparison (OR = 0.633, 95% CI: 0.446–0.899, *p* = 0.011, *I*^2^ = 57.7%), recessive model (OR = 0.632, 95% CI: 0.478–0.837, *p* = 0.001, *I*^2^ = 0%), and dominant model (OR = 0.588, 95% CI: 0.414–0.837, *p* = 0.003, *I*^2^ = 62.9%), but not in over-dominant (OR = 1.216, 95% CI: 0.944–1.567, *p* = 0.130, *I*^2^ = 34.1%). After Bonferroni correction, the associations remained significant for the allelic, homozygote, recessive, and dominant models.

For the association between G allele in *SCN1A* IVS5N+5 G>A polymorphism and risk of all febrile seizure, a significant protective effect was detected in the allelic model (OR = 0.690, 95% CI: 0.530–0.860, *p* = 0.001, *I*^2^ = 56.8%), homozygote comparison (OR = 0.503, 95% CI: 0.279–0.908, *p* = 0.023, *I*^2^ = 70.1%), heterozygote comparison (OR = 0.581, 95% CI: 0.460–0.733, *p* < 0.001, *I*^2^ = 0%), dominant model (OR = 0.543, 95% CI: 0.436–0.677, *p* < 0.001, *I*^2^ = 0%), and over-dominant (OR = 1.309, 95% CI: 1.061–1.615, *p* = 0.012, *I*^2^ = 0%), but not in recessive model (OR = 0.699, 95% CI: 0.394–1.241, *p* = 0.221, *I*^2^ = 74.5%). After Bonferroni correction, the associations remained significant for the allelic, heterozygote, and dominant models.

#### Epilepsy

For the association between G allele in *SCN1A* IVS5N+5 G>A polymorphism and risk of epilepsy without febrile seizure, a significant negative association was detected in the allelic model (OR = 0.882, 95% CI: 0.784–0.993, *p* = 0.038, *I*^2^ = 38.7%), recessive model (OR = 0.908, 95% CI: 0.478–0.837, *p* = 0.001, *I*^2^ = 0%), and dominant model (OR = 0.816, 95% CI: 0.716–0.930, *p* = 0.002, *I*^2^ = 0%), but not in homozygote comparison (OR = 0.799, 95% CI: 0.615–1.037, *p* = 0.091, *I*^2^ = 46.6%), heterozygote comparison (OR = 0.823, 95% CI: 0.716–0.945, *p* = 0.006, *I*^2^ = 0%), and over-dominant model (OR = 1.122, 95% CI: 0.948–1.329, *p* = 0.181, *I*^2^ = 40.8%). After Bonferroni correction, the associations remained significant for the recessive and dominant models.

For the association between G allele in *SCN1A* IVS5N+5 G>A polymorphism and risk of all epilepsies, a significant protective effect was detected in the allelic model (OR = 0.822, 95% CI: 0.750–0.902, *p* < 0.001, *I*^2^ = 59.3%), homozygote comparison (OR = 0.655, 95% CI: 0.515–0.832, *p* = 0.001, *I*^2^ = 66.9%), heterozygote comparison (OR = 0.780, 95% CI: 0.705–0.862, *p* < 0.001, *I*^2^ = 0%), recessive model (OR = 0.769, 95% CI: 0.625–0.947, *p* = 0.013, *I*^2^ = 67.6%), dominant model (OR = 0.743, 95% CI: 0.663–0.833, *p* < 0.001, *I*^2^ = 25.5%), and over-dominant (OR = 1.093, 95% CI: 1.001–1.193, *p* = 0.046, *I*^2^ = 8%). After Bonferroni correction, the associations remained significant for the allelic, homozygote, and dominant models.

The sub-group analysis indicated that this negative association with all epilepsies was found stronger among Indian (G vs. A: OR = 0.696, 95% CI: 0.530–0.915, *p* = 0.009 *I*^2^ = 75.2%) than in Caucasian (OR = 0.873, 95% CI: 0.792–0.962, *p* = 0.006, *I*^2^ = 0%) and Chinese populations (OR = 0.869, 95% CI: 0.797–0.947, *p* = 0.001, *I*^2^ = 0%). The negative association was observed to be stronger in studies using population-based control (G vs. A: OR = 0.752, 95% CI: 0.607–0.931, *p* = 0.009, *I*^2^ = 82.0%) than in studies using hospital-based controls (OR = 0.870, 95% CI: 0.807–0.937, *p* < 0.001 *I*^2^ = 0%).

#### Comparison Between Febrile Seizure and Epilepsy

The negative association was stronger in patients with pure febrile seizures than in patients with pure epilepsy for the allelic model (OR = 0.728, 95% CI: 0.600–0.884, *p* = 0.001, *I*^2^ = 15.5% vs. OR = 0.882, 95% CI: 0.784–0.993, *p* = 0.038, *I*^2^ = 38.7%) and dominant model (OR = 0.589, 95% CI: 0.443–0.783, *p* < 0.001, *I*^2^ = 0% vs. OR = 0.816, 95% CI: 0.716–0.930, *p* = 0.002, *I*^2^ = 0%).

## Discussion

### Summary of Major Findings

This systematic review and meta-analysis evaluated the association between the *SCN1A* IVS5N+5 G>A polymorphism and susceptibility to febrile seizure and epilepsy using the most updated data from 12 studies involving a total of 11,862 participants. We identified a significant negative association between the G allele in *SCN1A* IVS5N+5 G>A polymorphism and risk of different clinical subtypes of febrile seizure (including febrile seizure alone, febrile seizure with epilepsy, and all febrile seizures) and epilepsy (epilepsy without a febrile seizure and all epilepsies) using different genetics models, especially for allelic and dominant models. The negative association with all epilepsies was found to be stronger among Indian than in Caucasian and Chinese populations in studies using population-based control than using hospital-based controls. We also observed that the negative association was stronger in patients with febrile seizures than patients with epilepsy.

### Explanations of Findings and Relationship With Literature

The current study found a significant negative association between G allele in the *SCN1A* IVS5N+5 G>A polymorphism and risk of different of pure febrile seizure, febrile seizure with epilepsy, all febrile seizures, epilepsy without a febrile seizure, and all epilepsies. This association was detected for all clinical subtypes of febrile seizure and epilepsy by dominant models, indicating that the G allele in *SCN1A* IVS5N+5 G>A polymorphism was dominant in decreasing the risk of febrile seizure and epilepsy among children. Evidence suggested febrile seizure with epilepsy is commonly observed among family members with the mutations in *SCN1A* (30). Nevertheless, the mechanism behind is unknown. This is probably due to the fact that the neuronal membrane excitability is a closely regulated factor and that change in the function of the ion channel can result in the corresponding alternation in other channels (31). Some SNPs in the *SCN1A* gene have been detected frequently among children with an extremely severe type of epilepsy (32). Among these SNPs, emerging evidence shows that the IVS5N+5 G>A polymorphism plays an important role (18). This SNP determines the relative expression level of the alternative splicing proteins. The neonatal exon was preferentially translated in the early developmental time and was upregulated after febrile seizures among children (33) This SNP may influence the change of imbalance between membrane excitability and inhibition at different levels (15).

Heterogeneity is an important issue to be addressed as it may have an impact on the interpretation of the results. We observed that the levels of association were different between ethnicities, sources of control, and clinical subtypes of febrile seizure and epilepsy. The difference may be caused by the disparities in the prevalence of SNPs and association disequilibrium with other risk-associated SNPs between populations in different geographical regions. It was well-recognized that the disease may be caused by the integrations between genetics, host, and environmental factors (34). In addition to the difference in genetics, the environmental risk factors, including climate, culture, and pathogens may also be attributed to the heterogeneity of association observed (34). In the subgroup analysis by the source of control, we found that the negative association with risk of febrile seizure and epilepsy was stronger in studies using population-based control than using hospital-based control in allelic and dominant models. This is probably due to the fact that hospital-based controls were recruited within the hospital and may not be representative of the general population. The controls may include patients with other genetics-related diseases, which may have underestimated the effects of the investigated SNP on the risk of febrile seizure and epilepsy. We also observed that the negative association of G allele in *SCN1A* IVS5N+5 G>A polymorphism was stronger in the risk of seizure (including febrile seizure alone, febrile seizure with epilepsy, and all febrile seizures) than in epilepsy (epilepsy without a febrile seizure and all epilepsies). The mechanism behind this remains unknown. It is speculated that G allele in *SCN1A* IVS5N+5 G>A may play a protective role in the development of febrile seizure than in epilepsy. However, this phenomenon observed may need to be further investigated.

Although there were systematic reviews on a similar topic, some major limitations were found in the previous studies. The study by Tang et al. (35). investigated the association between *SCN1A* rs3812718 polymorphism and risk of epilepsy with febrile seizures and concluded that SCN1A rs3812718 polymorphism is a risk factor of epilepsy with febrile seizures and epilepsy among Caucasian but not in Indian and Chinese populations. However, the results had some limitations as only six studies were included in this study, limiting the statistical power to detect a positive association. The conclusions drawn from the subgroup analysis by ethnicities may be limited by the small sample size. Additionally, the quality of each study was not evaluated, which is an essential part of a systematic review. This study may have included some primary studies with low quality, resulting in a bias in the estimation of meta-analysis. Another study by Zhi et al. (36) also investigated the association between *SCN1A* rs3812718 polymorphism and the risk of epilepsy. They detected an association between *SCN1A* rs3812718 polymorphism and risk of epilepsy in the homozygote and dominant models, which is consistent with our findings. However, they did not analyze the association in Indian and Chinese populations, probably also limited by the small sample size of studies included (*n* = 8). The previous meta-analysis also did not include children with pure febrile seizures in the meta-analysis. It remained unknown whether this SNP was associated with the risk of pure febrile seizures based on the previous results, which is the primary outcome of interest in our study. Also, all previous studies only reviewed articles published in English which may have caused language bias.

### Strengths and Limitations

The current study is the most updated systematic review and meta-analysis on this topic including a large sample size of participants. We did not limit the period and language of publication in the literature search. This enabled the analysis on different clinical subtypes and subgroups of febrile seizure and epilepsy using different genetic models by the enhanced statistical power. The overall quality of the included studies was good when evaluated by the NOS quality assessment scales, indicating the reliability of the meta-analysis results. However, there were also some limitations in the studies. First, most of these studies used case–control design, and some studies recruited the controls from the hospitals. The selection process of cases and controls may vary across studies, and this may have caused selection bias. Second, the retrospective nature of the study design could not establish the cause-and-effect relationship between the SNPs and risk of disease. Third, the SNP–SNP interactions and epigenetics risk factors, including age, gender, family history, body temperature, pathogens, and severity of the diseases, lifestyle risk factors, and environmental risk factors were not always controlled in the primary studies. More studies with better methodological design are needed to explore the possible interactions between genetics, host, and environment on the development of febrile seizure and epilepsy. In addition, the limited total number of studies may have lowered the detection power of the current analysis. Future meta-analysis including more studies when available is needed to confirm our findings.

### Implications

The results of the current study have some implications for clinical practice and future research on this topic. The *SCN1A* IVS5N+5 G>A polymorphism has the potential to be the biomarker tested clinically to determine the risk of febrile seizure and epilepsy among children. Those tested with A allele in the *SCN1A* IVS5N+5 G>A polymorphism may have a higher risk of febrile seizure and epilepsy and need related preventive measures when having fever and viral infection. This SNP may have some implications for developing the treatment of febrile seizure and epilepsy. The results support the evidence that *SCN1A* plays an important role in the occurrence of febrile seizure and epilepsy. However, it remains unclear why this association varied between different ethnicities and different clinical subtypes of seizure and epilepsy. In addition to IVS5N+5 G>A polymorphism, other SNPs in *SCN1A* and other genes may also have an important impact on the risk of febrile seizure and epilepsy, which requires our further investigation. Future research can also explore the possible interactions between generics, host, and environment on the development of febrile seizure, epilepsy, and drug-resistant epilepsy.

## Data Availability Statement

The original contributions presented in the study are included in the article/Supplementary Material, further inquiries can be directed to the corresponding author/s.

## Author Contributions

JH and GD designed the study and wrote the manuscript. JH, HL, and GD searched the literature and extracted the data. JM and GL performed the quality assessment and data analysis. PL and FX revised the manuscript. All authors approved the submission of this manuscript.

## Conflict of Interest

The authors declare that the research was conducted in the absence of any commercial or financial relationships that could be construed as a potential conflict of interest.

## Abbreviations

CI: confidence interval
HWE: Hardy–Weinberg equilibrium
OR: odds ratios
SNP: single nucleotide polymorphisms.
