# Supplementary material for: SCN1A IVS5N+5 G>A Polymorphism and Risk of Febrile Seizure and Epilepsy: A Systematic Review and Meta-Analysis
Source: Front Neurol. 2020 Dec 17;11:581539. doi: 10.3389/fneur.2020.581539 (PMC7773848; doi:10.3389/fneur.2020.581539)
Supplement: Supplementary file 1 [file Image_1.PDF]

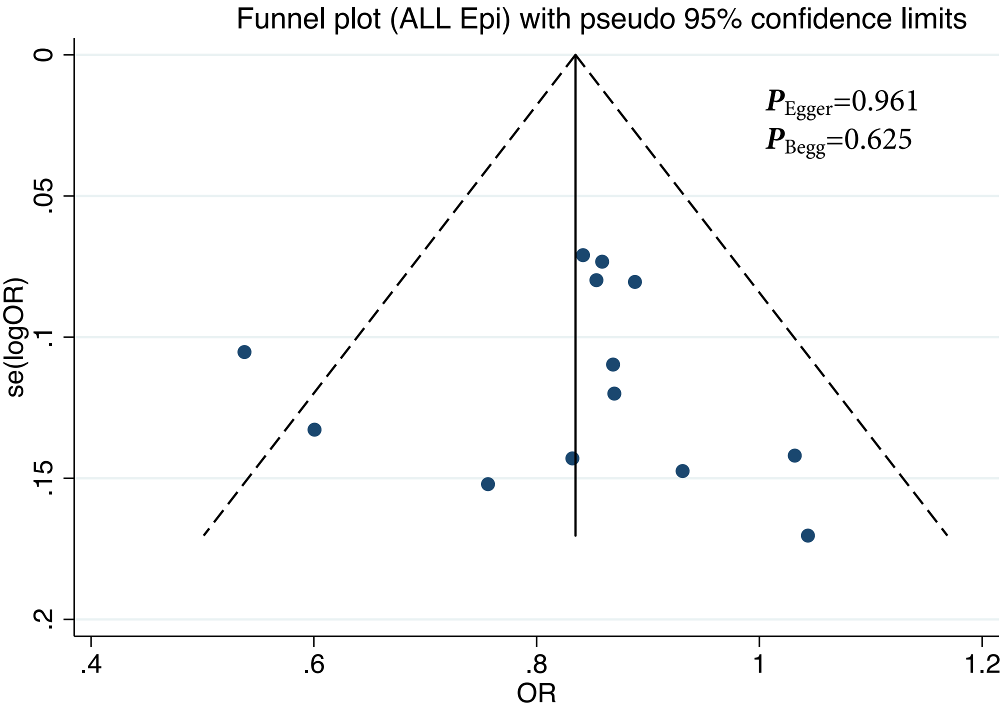
Funnel plot (ALL Epi) with pseudo 95% confidence limits
P_{Egger}=0.961
P_{Begg}=0.625
se(logOR)
0
.05
.1
.15
.2
.4
.6
.8
1
1.2
OR

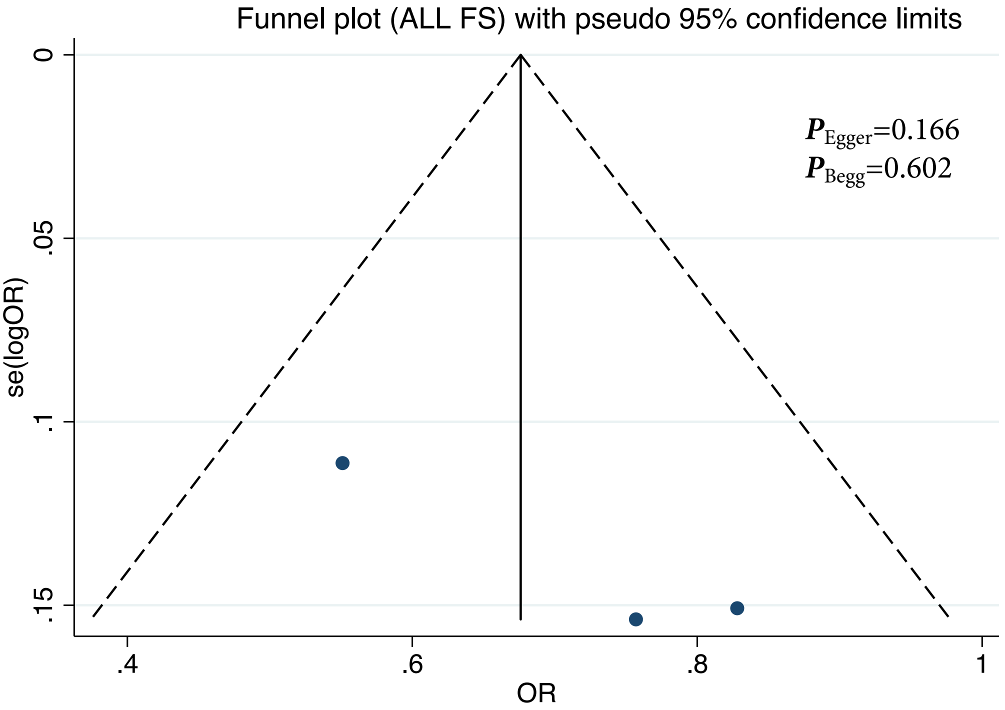

Funnel plot (ALL FS) with pseudo 95% confidence limits
P_{Egger}=0.166
P_{Begg}=0.602
se(logOR)
0
.05
.1
.15
.4
.6
.8
1
OR

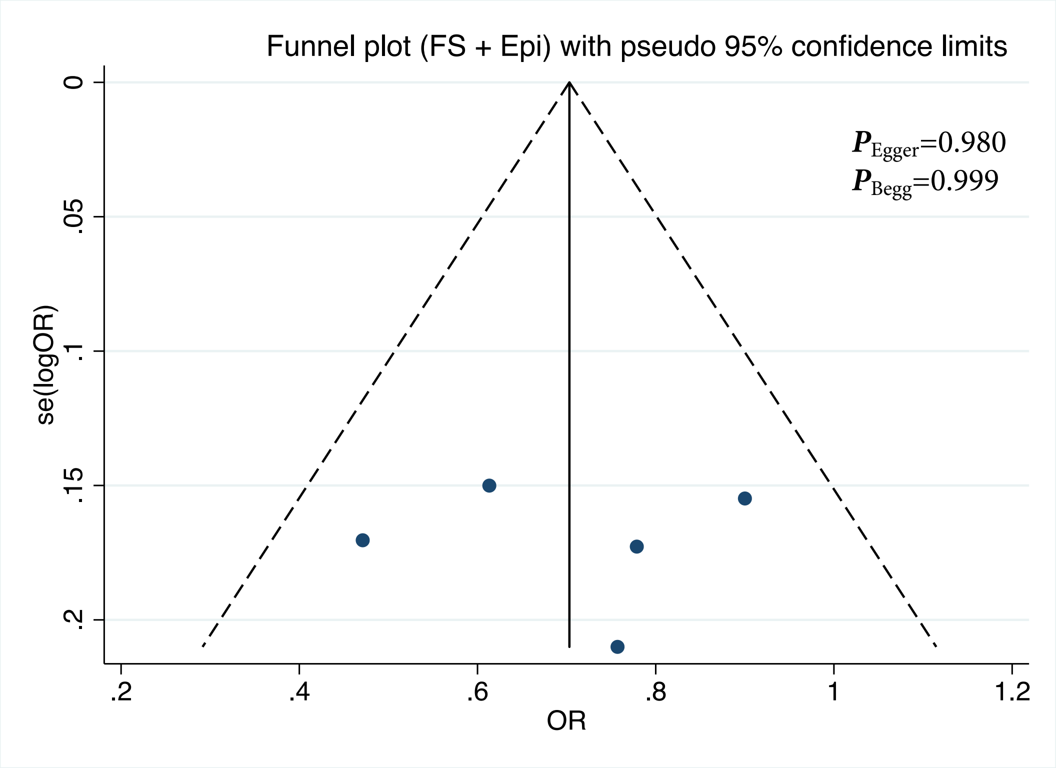

Funnel plot (FS + Epi) with pseudo 95% confidence limits
P Egger=0.980
P Begg=0.999
se(logOR)
0
.05
.1
.15
.2
.2
.4
.6
.8
1
1.2
OR

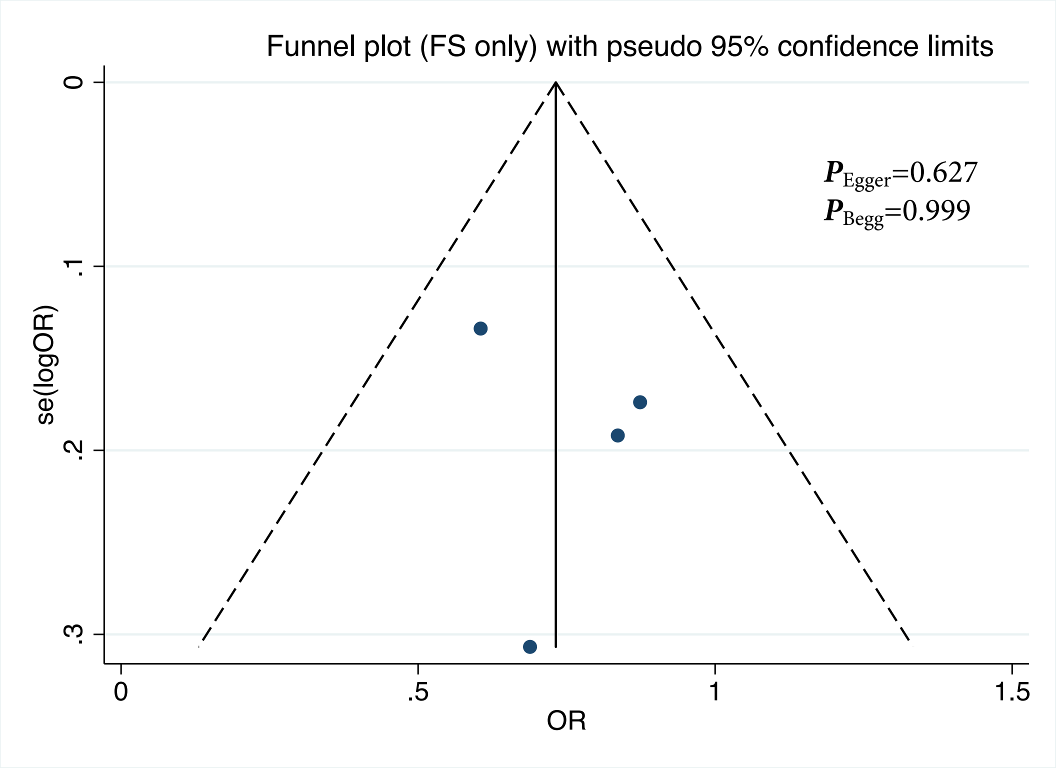
Funnel plot (FS only) with pseudo 95% confidence limits
P_{Egger}=0.627
P_{Begg}=0.999
se(logOR)
0
.1
.2
.3
0
.5
1
1.5
OR

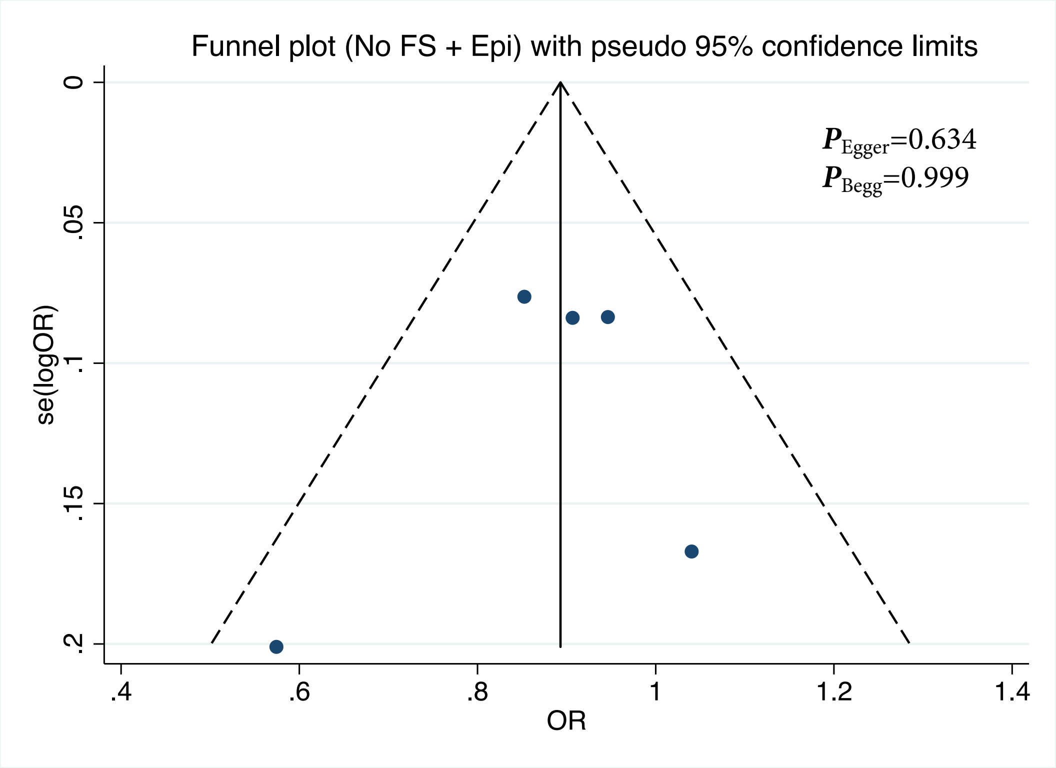

Funnel plot (No FS + Epi) with pseudo 95% confidence limits
P_{Egger}=0.634
P_{Begg}=0.999
0
.05
.1
.15
.2
se(logOR)
.4
.6
.8
1
1.2
1.4
OR
